# Positive non-linear capacitance: the origin of the steep subthreshold-slope in ferroelectric FETs

**DOI:** 10.1038/s41598-019-51237-2

**Published:** 2019-10-18

**Authors:** Md Nur K. Alam, P. Roussel, M. Heyns, J. Van Houdt

**Affiliations:** 1 Interuniversity Microelectronics Centre (imec), 75 Kapeldreef, B-3001 Leuven, Belgium; 2 Department of Materials Engineering, K. U. Leuven, Kasteelpark Arenberg 44 - box 2450, 3001 Leuven, Belgium; 3 ESAT, K. U. Leuven, Kasteelpark Arenberg 10 - box 2444, 3001 Leuven, Belgium

**Keywords:** Electronic devices, Electrical and electronic engineering, Electronic and spintronic devices

## Abstract

We show that the non-linear positive capacitance (PC) of ferroelectrics (FE) can explain the steep subthreshold-slope (SS) observed in FE based MOSFETs and often attributed to the existence of a negative capacitance in FE capacitors. Physically attainable and unattainable regions of the S-shape curve used in the negative capacitance theory are investigated by self-consistently solving Landau-Khalatnikov and Maxwell equations and by experimental validation. Finally, the conditions for attaining a steep SS in FE based MOSFETs assuming only positive capacitances are discussed.

## Introduction

In the non-degenerate limit, the charge carriers in a MOSFET follow the Boltzmann distribution. This leads to a fundamental limit in the steepness of the I-V characteristic in the subthreshold regime, which is 60mV/dec at room temperature. Breaking this limit has been the topic of intense investigations over the last years as it would allow producing CMOS circuits that consume less power. The Negative Capacitance (NC) based FET in which the regular gate oxide would be replaced by a ferroelectric (FE) oxide was proposed some time ago to beat this “Boltzmann tyranny”^1^. This concept assumed that a *quasi-static* (*QS*) *NC* can occur in FE based dielectric stacks, and a Landau based formalism was used to predict its existence^1^. The theory predicted a surface potential amplification upon stabilization of the quasi-static NC, which is quantified by the body factor ‘*m*’. Subsequently, sub 60mV/dec behavior in limited regions of the FE MOS subthreshold regime has been experimentally demonstrated as well as explained by QSNC theory in various works^2–6^. In addition, direct observation of NC was reported in^7^ as well.

However, this is still a topic of scientific controversy. After the first direct demonstration of NC, there have been several papers giving alternative explanations for the experimental observations^8–11^. The opposing school claims the phenomenon is attributed to a *dynamic effect* of the polarization switching that causes a snap-back in the voltage across the FE layer. The steep-slope behavior observed in MOS devices is also explained from this concept^11,12^. In^10^ authors have shown that all experimental observations can be explained both from a Landau model (assuming QSNC exists) as well as a Miller model (assuming QSNC does not exist). Until now there has been no scientific falsification of either of these approaches. While recently the existence of steady-state NC (stabilized QSNC) was claimed again^13^, nevertheless it doesn’t completely resolve the controversy. The QSNC is observed only at the domain boundaries. This is a very local effect within the FE material. The QSNC region is surrounded by non-NC regions which makes the overall capacitance of the FE thin-film positive, which means it impossible to see the NC effect from the outside.

In this paper, we resolve the issue and demonstrate that the experimental observations can also be described by taking into account the non-linearity of the capacitance which is typical for ferroelectric materials. It is theoretically and experimentally demonstrated that this straightforward concept provides a good description of the physical reality without having to invoke the concept of negative capacitance.

## Motivation for Non-Linear Capacitance Concept

The behavior of a general capacitor structure can be described by its absolute capacitance, given by *C* = *Q/V*, and the differential capacitance, given by *C*_*dif*_ = *dQ/dV*. In non-linear capacitors, a negative differential capacitance can occur. To explain the importance of non-linear effects we first briefly discuss the microscopic polarization of FE materials and explore the limits of the generally accepted Landau theory.

Each unit cell of such a material is polarized with a dipole moment *P*_*dp*_ = *P*_0_ and experiences a local electric field *E*_*local*_ that comes from the sum of the electric field created by the surrounding dipoles, given by *E*_*dipole*_ = *ηP*_*dp*_/*ε*_0_, and the externally applied electric field *E*. Here *η* is a lattice dependent parameter, *P*_*dp*_ is the dipole moment of each individual dipole and *ε*_0_ is the vacuum permittivity. For example, in a cubic crystal $\eta =1/(3{a}^{3})$ where *a*^3^ is the volume of the unit cell^14^. Assuming an infinite series of dipoles oriented in the same direction *P*_*dp*_ increases further from *P*_0_ in response to *E*_*local*_. This in turn increases *E*_*dipole*_. This positive feedback would infinitely increase *P*_*dp*_. Feynman already noted^14^ that the mathematical consequence of such a ‘catastrophic’ event would imply that the dielectric permittivity of the material *ε*(= *ε*_0_*ε*_*r*_) with *ε*_0_ the vacuum permittivity and *ε*_*r*_ the relative permittivity) would become negative. However, a singularity occurs before going into this negative *ε* regime. The general theory is only valid in the regime before this point is reached and in reality non-linear effects take over in FE materials before this singularity is reached. The positive feedback mechanism before reaching this singularity thus causes a self-sustained polarization state in the FE^14^. The Landau theory^15^ describes this self-sustained polarization from the thermodynamic perspective, by considering the free energy of the FE material given by:

1 $$U={P}^{2}+\beta {P}^{4}-EP$$

Here *α* and *β* are phenomenological fitting parameters and *P* is the vectorial sum of *P*_*dp*_ from all unit cells. The free energy landscape of the system possesses two energetically degenerate minima due to spontaneous symmetry breaking. Without an external field, the system stabilizes at either of these minima with a remnant polarization ±*P*_*r*_. When an electric field is applied the *P* of the system increases until *E* reaches the coercive field *E*_*c*_, at which point the polarization switches from −*P*_*r*_ to +*P*_*r*_ or vice-versa. At steady-state conditions the *E* − *P* relation can be obtained by minimizing *U* with respect to *P* resulting in^15^:

2 $$E=2\alpha P+4\beta {P}^{3}$$

**Figure 1:**
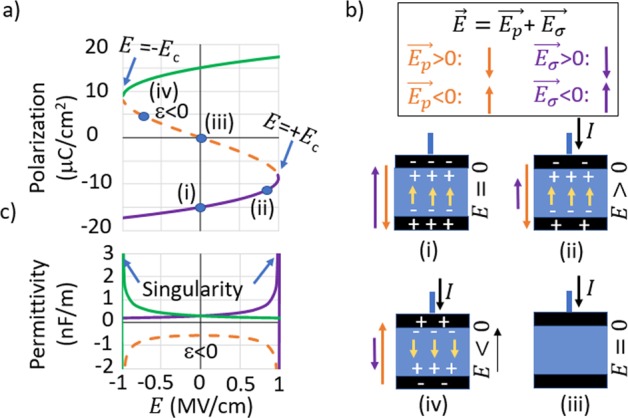
Using the Landau parameters: *α* = −8.119 × 10^8^ C^−2^Jm, *β* = 6.343 × 10^10^ C^−4^Jm^5^_,_ (**a**) P-E curve (or S-curve) given by equation ‘2’ (solid line) and assumed switching path in the NC model (dotted line) (**b**) the orientation of electric field and polarization on the four different regimes of the S-curve, (**c**) the dielectric permittivity of the ferroelectric material calculated from the slope of the P-E curve in the various regimes.

This equation describes the equilibrium part of the characteristic polarization curve of FE materials, shown by the solid line in Fig. 1(a). The dotted line in this figure is the switching path that is assumed in the theoretical models that support the claims of a negative capacitance^1^. This assumption results in a typical S-shape curve.

On the S curve in Fig. 1(a) we mark four regions ‘i’, ‘ii’, ‘iii’ and ‘iv’. During switching polarization changes from −Pr to +Pr which results in an external current flow to the FE in the positive direction. The net electric field *E* has two components: field created by the polarization *E*_*p*_ and by the surface charge *E*_σ_. In region ‘i’, ‘ii’ and ‘iii’ $E\ge 0$ while $I > 0$. But in region ‘iv’ located in the 2^nd^ quadrant of the *P* − *E* curve, $E < 0$ while $I > 0$, i.e., current flows against the electric field as depicted in Fig. 1(b). As this cannot happen while charging a capacitor, traversing the full S-curve during switching is not possible. In other words: in the 2^nd^ quadrant both *C*_*dif*_ < 0 and *C* < 0. The same contradiction is found in the 4^th^ quadrant of Fig. 1(a) when the polarization switches from +Pr to −Pr.

To investigate this further, one can calculate the dielectric permittivity from this curve, as shown in Fig. 1(c). As expected the ε calculated from the equilibrium part of the curve, indicated by the solid lines, show a singularity at the validity limit of Eq. 2. Although recently the full traversal of the S-curve was reported in a ferroelectric-dielectric stack^16^, authors noted that it disappears if they allow multiple domains to form. As a single domain ferroelectric is very hard to realize, most observation attempts failed^17^. It is also noteworthy that in^16^ authors started from $-{P}_{r}$ state (PC region), managed to traverse the full S-curve (i.e., NC region) and then reaching +*P*_*r*_ state (another PC region). Interestingly as they removed the voltage the system spontaneously switched back to the initial state. Authors claimed the 2^nd^ PC region is unstable. However this should not be happening according to QSNC theory (Landau picture), from which they attempted to explain the findings, thus leaving some unresolved issues in their work.

It has been previously suggested that a FE system can be stabilized on this S-curve and can be harnessed as a steady-state/equilibrium phenomenon (*QSNC*). This was counteracted by other studies claiming that the results presented in^7^ are not a part of the S-curve, but rather a snap-back of the voltage attributed to the *dynamic switching effects* in FE materials, including effects like nucleation and propagation. In the following section, we examine the predictions from both model types.

## Experimental

QSNC theory predicts the existence of NC which is intrinsically unstable. To experimentally observe this, it needs to be stabilized first. In a standard *P* − *E* measurement, no stabilization mechanism is present. Consequently, such measurement always shows a hysteretic *P* − *E* characteristic. In addition, the standard technique measures *P*(*E*), *E* being the independent/control variable. To be able to observe the S-curve, it is necessary to let *E* evolve as a function of *P*, i.e., *E*(*P*) measurement is needed. We implemented this *E*(*P*) measurement scheme. In addition, our scheme has inherent stabilization mechanism that should let us observe the S-curve predicted by the QSNC theory. In the following section, we show how the predictions from different models deviate and try to validate them against experimental results.

### Quasi-static NC concept

The NC can be stabilized by putting a non-ferroelectric capacitor in series with the FE. In such a series combination, the electric field seen by the FE is given by-

3 $$E=\frac{\frac{{{\epsilon }}_{0}{{\epsilon }}_{DE}}{{t}_{DE}}V-P}{{{\epsilon }}_{0}[{{\epsilon }}_{FE}+{{\epsilon }}_{DE}\frac{{t}_{FE}}{{t}_{DE}}]}$$

**Figure 2:**
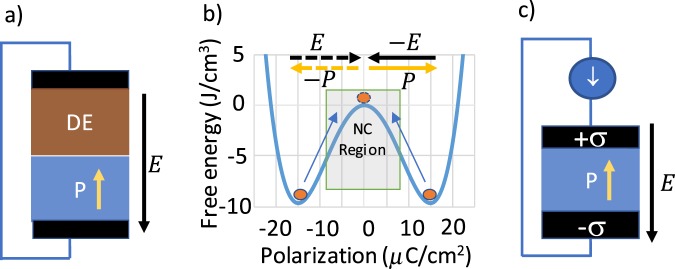
(**a**) Creation of depolarization field in ferroelectric-dielectric stack, (**b**) depolarization field acts as a stabilization mechanism of the negative capacitance in the ferroelectric, (**c**) a method of obtaining depolarization field by controlling the surface charge density.

Here ${{\epsilon }}_{FE(DE)},\,\,{t}_{FE(DE)}$, are relative permittivity and thickness of ferroelectric (non-ferroelectric) respectively, ${{\epsilon }}_{0}$ is the vacuum permittivity, *P* is the polarization in the FE and *V* is the applied voltage across the stack. When the voltage across the stack is zero as shown in Fig. 2(a), Eq. (3) implies the electric field is given by $E=-\,P/{{\epsilon }}_{0}[{{\epsilon }}_{FE}+{{\epsilon }}_{DE}\frac{{t}_{FE}}{{t}_{DE}}]$. The negative sign indicates that the electric field acts against the polarization. Such field is called the depolarization field^18^. Depolarization field pushes the polarization state from the minima towards the top of the Landau free energy landscape. Consequently it acts as a stabilization mechanism that keeps the FE in the NC region as shown in Fig. 2(b).

Note that this is not the only way to establish a depolarization field. The electric field inside a FE arises from the surface charge density $\sigma$ supplied from an external source as shown in Fig. 2(c). According to Maxwell’s equation:

4 $$\sigma ={\varepsilon }_{0}E+{P}_{pe}+P$$

*P*_*pe*_ comes from the paraelectric polarization of the FE material attributed to the movement of electron cloud in the material in response to the electric field. The paraelectric contribution is accounted for by the background dielectric constant *ε*_*r*(*pe*)_. From this equation we obtain:

5 $$E=\frac{\sigma -P}{{\varepsilon }_{0}{\varepsilon }_{r(pe)}}$$

It is clear that Eqs (3) and (5) are equivalent. Therefore, instead of connecting a series capacitor the NC can be stabilized by controlling the surface charge of the FE. Additionally in this scheme *E* becomes a dependent variable which is necessary to observe any S-curve.

In order to get the quantitative description of the polarization dynamics in the *σ*-controlled scheme (Fig. 2c) we solve the Landau- Khalatnikov (LK) equation^19^:

6 $$M\frac{{d}^{2}P}{d{t}^{2}}+L\frac{dP}{dt}=F$$

Here *M* is an inertia factor, *L* a viscosity factor and *F* is the force acting on the system given by:

7 $$F=-\,\frac{dU}{dP}=-\,(2\alpha P+4\beta {P}^{3})+E$$

**Figure 3:**
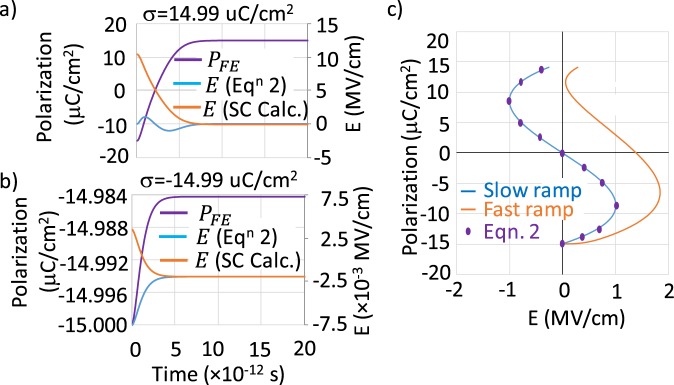
Polarization and electric field dynamics obtained by self-consistently solving Maxwell and Landau Khalatnikov equation, and compared against Eq. (2) for a step-function input of the surface charge (**a**) *σ* = +14.99 μC/cm^2^ (**b**) *σ* = −14.99 μC/cm^2^. (**c**) *P* − *E* relation obtained from self-consistent (SC) calculation when *σ* is varied from −14.99 to +14.99 μC/cm^2^ at different ramp rate and from Eq. (2). Landau parameters used in the simulation are: *α* = −8.119 × 10^8^ C^−2^ Jm, *β* = 6.343 × 10^10^ C^−4^ Jm^5^, *ε*_*r*(*pe*)_ = 22.

For a *σ* controlled NC stabilization mechanism we solve (5), (6) and (7) self-consistently. We assume initially the system is in −*P*_*r*_ state. When a surface charge *σ* is instantly supplied to the capacitor plates (step-function input), the polarization and electric field change in response as shown in Fig. 3(a,b). After an initial transient, the *E* obtained from the self-consistent solution converges to the results from the steady state Eq. (2). If we ramp up the *σ* then the *P* − *E* relation depends on the ramp rate. When the ramp rate is slower than the switching speed of the polarization, according to LK theory, one would attain a NC stabilization and the *P* − *E* curve would traverse the S-curve as shown in Fig. 3(c).

**Figure 4:**
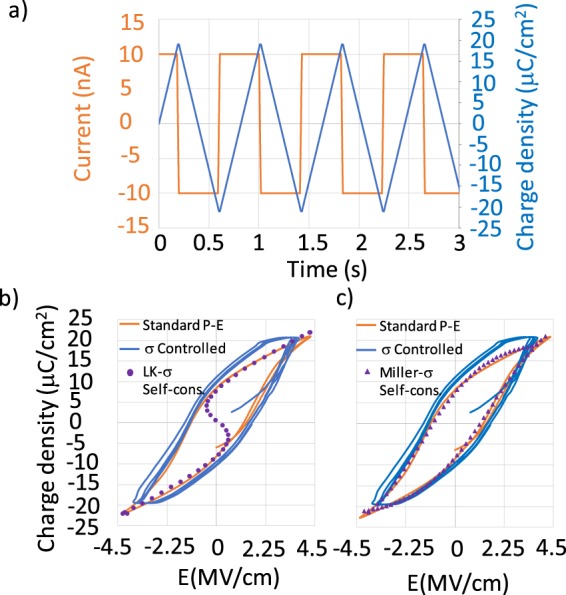
(**a**)Wave form of current and resultant surface charge density *σ* supplied to a FE capacitor from a current source that is used to measure *P* − *E* relation using *σ* as the independent variable (**b**) Comparison of the used method against standard *P* − *E* measurement as well as *σ* controlled LK simulation. Landau parameters used in the simulation are: *α* = −8.119 × 10^8^ C^−2^Jm, *β* = 6.343 × 10^10^ C^−4^Jm^5^, *ε*_*r*(*pe*)_ = 22, (**c**) Comparison of the measurements against Miller model.

To check the theoretical prediction against experiment we fabricate 8 nm thick 100 × 100 μm^2^ Al doped HfO_2_ Metal-Insulator-Metal (MIM) capacitors in which TiN is used as top and bottom electrode. The doping along with the strain produced by the metal electrodes stabilizes the HfO_2_ in a non-centrosymmetric orthorhombic phase. Consequently the MIM capacitor becomes FE. We push a constant current *i* to the FE capacitor plate (Fig. 2c). The surface charge density on the capacitor plate, given by $\sigma (t)=\frac{1}{A}{\int }_{0}^{t}idt$, increases linearly with time. By altering the direction of the current flow, a sawtooth *σ*(*t*) profile is obtained as shown in Fig. 4(a). While we ramp the *σ* the voltage developed across the FE is monitored. The *P* − *E* curve obtained with this method doesn’t traverse the S-curve in reality, instead it is similar to those obtained from the standard *P* − *E* measurement, as shown in Fig. 4(b). Using this measurement technique no stabilization of NC in the HfO_2_ FE capacitor can be observed, despite of the fact that our technique possesses all the prerequisites claimed by QSNC theory. This strongly suggests that the NC effect is a consequence of the assumption that the switching behavior of FE materials can be described by the steady state Eq. (2) and is supposed to follow the dotted line in Fig. 1(a). This assumption also goes beyond the claims of Landau who pointed out that Eq. (2) only describes the stable region, indicated by the solid line in Fig. 1(a)^15^.

### Non-linear (dynamic) capacitance concept

In order to clarify this point further, more insight is needed into the switching behavior of FE materials since the interpolation path between the two steady-state conditions is not enough. As switching is a dynamic condition, various time-dependent effects will be present and it cannot be readily assumed that a steady-state formalism can capture the intricate details of this switching process. Furthermore, in real materials domain walls and domain wall motion will also play a significant role and directly affect this transition regime^8,9,20^.

As an overall observation no voltage snap back could be observed in any of our *σ* controlled measurements. In our case no effort was made to fine-tune the measurement conditions in order to experimentally observe the snap-back. Also the MIM capacitors used in this study have many domains that take part in the switching. When the energy involved with domain formation and domain boundaries are added to the free energy of the FE, the switching event shows reluctance in snapping back as it can find energetically more favorable bypasses^8^. It has been pointed out that even in the case of FE crystals that closely resemble single domain switching, a direct observation of the snap back requires specific measurement conditions which depend on the parasitics of the measurement system as well as the properties of the FE capacitor^21^. Adding external series resistance to change the time constant of the system response has been used to experimentally observe the snap-back behavior. The voltage across the FE did not reverse sign (to enter region iv in Fig. 1a) in those experiments^7^. In our measurements the switching of each single domain in the FE capacitor gets averaged out without snap-back, resulting in a smooth but non-linear charge-voltage characteristic.

Such non-linear charge-voltage characteristic is also captured in the Miller model where capacitance of the material is kept strictly positive, and given by^22^-

8 $$P(E)={P}_{s}\,tanh[\frac{E-{E}_{c}}{2{E}_{c}{\{ln(\frac{1+{P}_{r}/{P}_{s}}{1-{P}_{r}/{P}_{s}})\}}^{-1}}]$$

Here *P*_*r*_ and *P*_*s*_ are remanant polarization and saturation polarization respectively. As the Miller model can also

explain the voltage snap back under suitable measurement conditions (essentially by perceiving the dynamic effects)^10^, we check the predictions of the Miller model under our measurement conditions. For that we solve Eqs (5) and (8) self-consistently. As shown in Fig. 4(c) the measurement is in good agreement with the model’s prediction.

## Discussion

**Figure 5:**
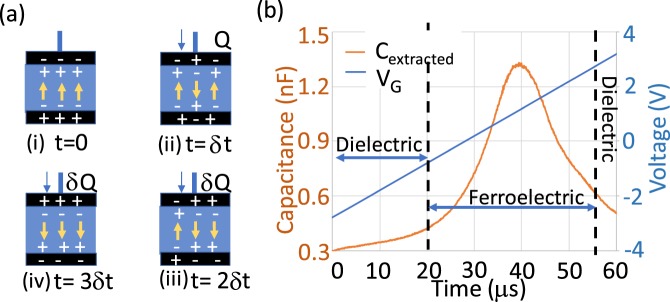
(**a**) Schematic illustration of the flipping of different dipoles in a FE capacitor during a voltage ramp with constant ramp rate and subsequent charge transfer from the voltage source (**b**) Time evolution of the dynamic capacitance.

From this we can understand the details of the non-linear charge-voltage characteristics of FE that can be translated into non-linear capacitance of a FE stack (*C*_*FE*_). Applying a voltage *V* over the dielectric creates an electric field *E* inside the FE material. If *E* is small the material shows a regular dielectric behavior, albeit with an absolute capacitance *C*_0_ that depends on the history of the material due to the possible presence of hysteresis. When *E* gets larger than the coercive field, the dipoles of the FE capacitor start flipping. This changes the local electric field and will trigger the neighboring dipoles to flip as well, resulting in a domain wall propagation^9^. To compensate for the effect of the flipping dipole some charge δ*Q* must flow to the plate of the capacitor from an external source in order to maintain the voltage across the capacitor. This is schematically illustrated in Fig. 5(a). In case the transfer of δ*Q* to the capacitor plate takes more time than that required for the flipping of the dipoles, the electric field inside the FE snaps back, such that Eq. (4) is satisfied at every instant^9,11^.

As a result, the relation between charge and voltage in a FE capacitor has a strong time dependence. More specifically, as the dipole flipping continues, the net charge flow $Q(t)={\sum }_{t=0}^{t}\delta Q$ increases with time even if the external voltage is kept constant. Consequently the absolute capacitance *C*_*FE*_(*t*) = *Q*(*t*)/*V* also becomes a function of time, but always remains positive. On the other hand, depending on the ratio of the time required to flip a dipole versus the time needed for compensating this by adding surface charge, the differential capacitance *C*_*FE(diff) *_(*t*) = *dQ*(*t*)/*dV* can become negative in a certain time window.

These effects can also be observed when a voltage ramp is applied to the FE capacitor. Figure 5(b) shows *C*_*FE*_ of the FE HfO_2_ MIM as extracted from the standard *P* − *E* measurement, i.e. with an increasing voltage ramp with constant ramp rate. It is clearly observed that at the point where the dipole flipping starts to occur, *C*_*FE*_ shows a strong non-linear increase^9^. When most of the dipoles have changed direction ${C}_{FE}$ drops and converges back to its initial value.

At this point the interesting question is whether such time-dependent capacitance behavior can provide steep slope operation in MOSFETs. The subthreshold slope (SS) is defined by:

9 $$SS={[\frac{{\log }_{10}{I}_{d}}{d{V}_{G}}]}^{-1}={[\frac{{\log }_{10}{I}_{d}}{d{\psi }_{s}}]}^{-1}{[\frac{d{\psi }_{s}}{d{V}_{G}}]}^{-1}=n\times m$$

**Figure 6:**
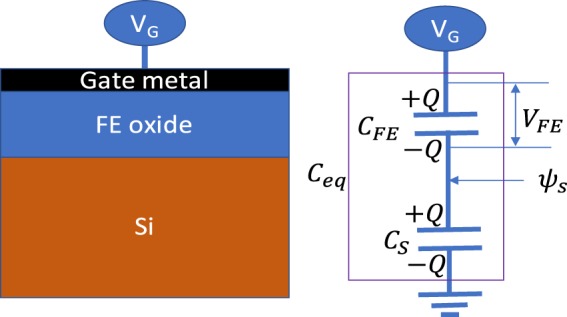
Ferroelectric MOS structure and its equivalent capacitor network.

Where *I*_*d*_ is the drain current, *V*_*G*_ the gate voltage and *ψ*_*s*_ the surface-potential. $n={[\frac{{\log }_{10}{I}_{d}}{d{\psi }_{s}}]}^{-1}=2.3\frac{kT}{q}$ is 60 mV/dec at room temperature and $m=\frac{d{V}_{G}}{d{\psi }_{S}}$ is the body factor. If the value of *m* becomes less than unity we get steep slope. The body factor *m* is calculated from the charge balance condition for a MOS structure shown in Fig. 6 as (appendix):

10 $$m=1+\frac{1}{{C}_{FE}}[\frac{d({C}_{S}{\psi }_{S})}{d{\psi }_{S}}-{V}_{FE}\frac{d{C}_{FE}}{d{\psi }_{S}}]\,$$

Where *C*_*S*_ is the capacitance of the semiconductor. Note that if both *C*_*S*_ and *C*_*FE*_ are assumed to be constant, this equation gets reduced to the usually applied formula $m=1+{C}_{S}/{C}_{FE}$^1^. However, in the case of an FE MOSFET, the gate capacitance *C*_*FE*_(*t*) is strongly non-linear and depends on time. It can be demonstrated that under typical conditions where the FE switching is very fast compared to gate bias ramp rate, *m* can go below unity and a steep slope will be observed during a certain part of the transistor switching (appendix). The reason behind this is that with increasing *V*_*G*_ the *Q* of the stack also increases. However, in a certain region of the voltage ramp the non-linear increase in *C*_*FE*_ can be sufficiently fast to disturb the charge balance of the MOS stack due to the limited supply current. As a response *V*_*FE*_ must decrease to restore this balance (appendix). As *V*_*FE*_ decreases while *V*_*G*_ continues to increase, *ψ*_*s*_ increases at a faster rate than *V*_*G*_, leading to ${[\frac{d{\psi }_{s}}{d{V}_{G}}]}^{-1} < 1$ (appendix). This demonstrates that one does not need to invoke the presence of a negative total capacitance to explain the steep SS in FE MOSFETs.

## Conclusion

In summary we have studied the capacitance of FE stacks to obtain more insight into the so-called negative capacitance effect. A charge controlled polarization measurement scheme is designed and implemented, that should traverse the full S-curve according to the prediction of Landau-Khalatnikov and Maxwell’s equations when solved self-consistently. Experimental results did not show any S-curve and from this, it is concluded that the FE capacitance is strongly non-linear and time dependent. The voltage snap-back effect in a ferroelectric-dielectric stack is related to the time required for polarization switching and the rate at which external charge can be delivered to the system. Depending on the ratio of these time constants the differential capacitance can become negative, although the total capacitance remains positive under all circumstances. Even under these considerations a steep subthreshold-slope in ferroelectric FETs can occur.

## Appendix

The charge balance requires Kirchhoff’s law to be satisfied in the capacitor stack shown in Fig. 6. It is written as:

$$\frac{d}{dt}({C}_{FE}{V}_{FE})=\frac{d}{dt}({C}_{S}{\psi }_{S})$$

Or,

$${V}_{FE}d{C}_{FE}+{C}_{FE}d{V}_{FE}=d({C}_{S}{\psi }_{S})$$

Putting ${V}_{FE}={V}_{G}-{\psi }_{S}$, and dividing by $d{\psi }_{S}$ we get

i $$\begin{array}{c}{V}_{FE}\frac{d{C}_{FE}}{d{\psi }_{S}}+{C}_{FE}\frac{d{V}_{G}}{d{\psi }_{S}}-{C}_{FE}\frac{d{\psi }_{S}}{d{\psi }_{S}}=\frac{d({C}_{S}{\psi }_{S})}{d{\psi }_{S}}\\ \,{C}_{FE}m={C}_{FE}+\frac{d({C}_{S}{\psi }_{S})}{d{\psi }_{S}}-{V}_{FE}\frac{d{C}_{FE}}{d{\psi }_{S}}\\ \,m=1+\frac{1}{{C}_{FE}}[\frac{d({C}_{S}{\psi }_{S})}{d{\psi }_{S}}-{V}_{FE}\frac{d{C}_{FE}}{d{\psi }_{S}}]\\ \,\,=\,1+\frac{1}{{C}_{FE}}[\frac{d{Q}_{S}}{d{\psi }_{S}}-{V}_{FE}\frac{d{C}_{FE}}{d{\psi }_{S}}]\end{array}$$

Here *Q*_*s*_ is the charge in the semiconductor channel.

ii $$\frac{d{C}_{FE}}{d{\psi }_{S}}={[\frac{d{V}_{G}-d{V}_{FE}}{d{C}_{FE}}]}^{-1}={[\frac{d{V}_{G}/dt}{d{C}_{FE}/dt}-\frac{d{V}_{FE}}{d{C}_{FE}}]}^{-1}$$

The rate of change of *C*_*FE*_ depends on the domain wall propagation speed. Once a domain nucleates at a given voltage *V*_*FE*_, it can propagate by transferring the energy stored in one dipole to the next via phonon vibration. The speed is given by^23^ -

iii $${v}_{propagation}\propto exp[\frac{kT}{U}{(\frac{{V}_{FE}}{{V}_{co}})}^{\vartheta }]$$

Here *kT* is thermal energy, *U* is the kinetic barrier for propagation, *V*_*co*_ is the critical voltage at which the process starts, $\vartheta$ accounts for any defect/imperfection existing in FE material. The process depends on *V*_*FE*_ only and may continue without any aid from *V*_*G*_. Therefore if *V*_*G*_ is kept constant when propagation starts *C*_*FE*_ will continuously increase causing ${V}_{FE}$ to decrease. The propagation will continue until *V*_*FE*_ becomes zero. This translates to the fact that rate of change of *C*_*FE*_ can be positive when the rate of change of *V*_*G*_ is zero, i.e., $d{C}_{FE}/dt > d{V}_{G}/dt$. When *C*_*FE*_ increases at a much faster rate than *V*_*G*_, i.e., $d{C}_{FE}/dt\gg d{V}_{G}/dt$; first term in ‘ii’ can be neglected. That gives:

iv $$\frac{d{C}_{FE}}{d{\psi }_{S}}=-\,\frac{d{C}_{FE}}{d{V}_{FE}}$$

To carry out the derivative of dynamic *C*_*FE*_ we need an empirical equation that fits with the extracted capacitance shown in Fig. 5(b). We take-

v $${C}_{FE}={C}_{0}{e}^{\mu t}$$

Where +*μ* gives the increasing part and −*μ* gives the decreasing part of the *C*_*FE*_(*t*). At some instant *t*, charge in the equivalent capacitor and the charge in the FE shown in Fig. 5 is also balanced, so we can write:

$$\begin{array}{c}\frac{{C}_{s}{C}_{0}{e}^{\mu t}}{{C}_{s}+{C}_{0}{e}^{\mu t}}{V}_{G}={C}_{0}{e}^{\mu t}{V}_{FE}\end{array}$$

Or,

vi $$\begin{array}{c}{V}_{FE}=\frac{1}{1+\frac{{C}_{0}}{{C}_{s}}{e}^{\mu t}}{V}_{G}={V}_{G}\frac{{C}_{s}}{{C}_{0}}{e}^{-\mu t}\end{array}$$

Here we see *V*_*FE*_ decreasing when *C*_*FE*_ is increasing. Furthermore from ‘v’ and ‘vi’ we find-

$$\frac{d{C}_{FE}}{d{V}_{FE}}=\frac{d{C}_{FE}}{dt}\frac{dt}{d{V}_{FE}}=\mu {C}_{FE}{[-\mu {V}_{FE}+(\frac{{C}_{s}}{{C}_{0}}\frac{d{V}_{G}}{dt}+\frac{{V}_{G}}{{C}_{0}}\frac{d{C}_{s}}{dt}){e}^{-\mu t}]}^{-1}$$

Neglecting the exponentially decaying term we get:

vii $$\frac{d{C}_{FE}}{d{V}_{FE}}=-\,\frac{{C}_{FE}}{{V}_{FE}}$$

To what extent this approximation is true is subject to experimental determination. From ‘iv’ and ‘vii’ we get:

viii $$\frac{d{C}_{FE}}{d{\psi }_{S}}=\frac{{C}_{FE}}{{V}_{FE}}$$

Finally combining ‘i’ and ‘viii’ we get:

$$m=1+\frac{1}{{C}_{FE}}[{C}_{s}({Q}_{s})-{C}_{FE}]=\frac{{C}_{s}({Q}_{s})}{{C}_{FE}}=\frac{{C}_{s}({Q}_{s})}{{C}_{0}}{e}^{-\mu t}$$

If the gate bias sweep is given by ${V}_{g}=\upsilon t$ then the body factor is given by-

ix $$m=\frac{{C}_{s}({Q}_{s})}{{C}_{0}}{e}^{-\frac{\mu }{\upsilon }{V}_{g}}$$

If the ratio of ${C}_{s}({Q}_{s})$ to *C*_0_ is already close to unity, and rate factor of the dynamic *C*_*FE*_ is high enough, i.e., $\mu  > \upsilon$ then *m* can go below unity. Equation ‘ix’ suggests larger *C*_0_ is preferred to get smaller *m*; to make *C*_0_ larger, one must reduce the thickness of FE. Doing so will lead toward loss of ferroelectricity as well as the dynamic *C*_*FE*_ property. Thereby it imposes a major trade-off in the design of steep-slope FE-FET.

## References

[1] Salahuddin S, Datta S (2008). Use of Negative Capacitance to Provide Voltage Amplification for Low Power Nanoscale Devices. Nano Letters..

[2] Ko E, Lee JW, Shin C (2017). Negative Capacitance FinFET With Sub-20-mV/decade Subthreshold Slope and Minimal Hysteresis of 0.48 V. IEEE Electron Device Letters..

[3] Zhou J (2017). Ferroelectric Negative Capacitance GeSn PFETs With Sub-20 mV/decade Subthreshold Swing. IEEE Electron Device Letters..

[4] Si M (2018). Steep-slope hysteresis-free negative capacitance MoS2 transistors. Nature Nanotechnology..

[5] J. Zhou, *et al*, Ferroelectric HfZrOx Ge and GeSn PMOSFETs with Sub-60 mV/decade subthreshold swing, negligible hysteresis, and improved Ids, Ein *IEEE International Electron Devices Meeting* (*IEDM*), (2016).

[6] P. Sharma, *et al*, Impact of total and partial dipole switching on the switching slope of gate-last negative capacitance FETs with ferroelectric hafnium zirconium oxide gate stack, In *Symposium on VLSI Technology*, (2017).

[7] Khan AI (2015). Negative capacitance in a ferroelectric capacitor. Nature Materials..

[8] S. J. Song *et al*. Alternative interpretations for decreasing voltage with increasing charge in ferroelectric capacitors, *Nature Scientific Reports*, vol. 6, no. 20825, (2016). 10.1038/srep20825 PMC4750000 26864751

[9] J. Van Houdt & P. Roussel, Physical model for the steep subthreshold slope in ferroelectric FETs, *IEEE Electron Device Letters*, vol. 39, no. 6, pp. 877–880, (June 2018).

[10] Saha AK, Datta S, Gupta SK (2018). Negative capacitance in resistor-ferroelectric and ferroelectric-dielectric networks: Apparent or intrinsic?. Journal of Applied Physics.

[11] B. Obradovic, T. Rakshit, R. Hatcher, J. A. Kittl &M. S. Rodder, Ferroelectric Switching Delay as Cause of Negative Capacitance and the Implications to NCFETs, In *IEEE Symposium on VLSI Technology*, (2018).

[12] H. Wang, *et al*, New Insights into the Physical Origin of Negative Capacitance and Hysteresis in NCFETs, In *IEEE International Electron Devices Meeting* (*IEDM*), (2018).

[13] Yadav AK (2019). Spatially resolved steady-state negative capacitance. Nature..

[14] Feynman, R. P., Leighton, R. B. & Sands, M., Inside Dielectrics, In *The Feynman Lectures on Physics: Mainly Electromagnetism and Matter*,*Volume 2*, California Institute of Technology, pp. 11.1–11.11.

[15] Landau, L. D. & Lifshitz, E., Ferroelectrics, In *Electrodynamics of Continuous media*, *second edition*, Pergamon Press, pp. 77–83.

[16] Hoffmann M (2019). Unveiling the double-well energy landscape in a ferroelectric layer. Nature..

[17] Z. Liu, M. A. Bhuiyan & T. P. Ma, A Critical Examination of ‘Quasi-Static Negative Capacitance’ (QSNC) theory, In *IEEE International Electron Devices Meeting* (*IEDM*), (2018).

[18] Ma T, Han J-P (2002). Why is nonvolatile ferroelectric memory field-effect transistor still elusive?. IEEE Electron Device Letters..

[19] Ricinschi D (1998). Analysis of ferroelectric switching in finite media as a Landau-type phase transition. J. Phys. Condens. Matter..

[20] Zubko P (2016). Negative capacitance in multidomain ferroelectric superlattices. Nature..

[21] X. Li, T. Nishimura & A. Toriumi, Direct measurement of internal potential in ferroelectric/paraelectric stack for studying Negative Capacitance effects, In *2018 International Conference on Solid State Devices and Materials*, *2018*, *pp883–884*, Tokyo, 2018.

[22] Miller SL, Schwank JR, Nasby RD, Rodgers MS (1991). Modeling ferroelectric capacitor switching with asymmetric nonperiodic input signals and arbitrary initial conditions. Journal of Applied Physics..

[23] Boddu, V., Endres, F. & Steinmann, P., Molecular dynamics study of ferroelectric domain nucleation and domain switching dynamics, *Nature Scientific Reports* 7, 806 (2017). 10.1038/s41598-017-01002-0 PMC5429757 28400598

